# Supplementary material for: Identification of an insect-produced olfactory cue that primes plant defenses
Source: Nat Commun. 2017 Aug 24;8:337. doi: 10.1038/s41467-017-00335-8 (PMC5569085; doi:10.1038/s41467-017-00335-8)
Supplement: Supplementary file 1 — Supplementary Information [file 41467_2017_335_MOESM1_ESM.pdf]

Description of Supplementary Files

File Name: Supplementary Information
Description: Supplementary Tables.

File Name: Peer Review File

Supplementary Table 1

Individual comparisons for single compound experiments

	**Leaf tissue removed (mm^2)**		**JA Concentration ($ng\ g^{-1}$)**	
**Treatment Comparisons**	**Difference in Means**	***P*-value**	**Difference in Means**	***P*-value**
Control / *Eurosta*	414.8	0.0009*	-168.8	0.004*
Control / *E,S*-Conophthorin	369.1	0.01*	-163.8	0.008*
Control / 1-Nonanol	85.3	0.95	-47.1	0.92
Control / Spiromix	232.8	0.18	-59.5	0.45
Eurosta / *E,S*-Conophthorin	-45.7	0.88	5.0	0.99
Eurosta / 1-Nonanol	-329.5	0.006*	121.7	0.03*
Eurosta / Spiromix	-182.3	0.18	109.3	0.19
E,S-Conophthorin / 1-Nonanol	-283.8	0.05*	116.7	0.05*
E,S-Conophthorin / Spiromix	-136.6	0.67	104.3	0.28
1-Nonanol / Spiromix	147.2	0.53	-12.4	0.90

Tukey post hoc multiple comparisons from the feeding assay and jasmonic acid induction experiment with individual compounds from the male *E. solidaginis* volatile emission (*n* = 6). Control = plants exposed to dichloromethane solvent, *Eurosta* = exposure to natural *E. solidaginis* emission, *E,S*-Conophthorin = exposure to pure *E,S*-conophthorin, 1-Nonanol = exposure to pure 1-nonanol, Spiromix = exposure to a mix of (2*E*) and (2*Z*)-2-methyl-1,6-dioxaspiro[4.5]decane). The "Difference in Means" values are for non-transformed data. Statistical analyses for leaf tissue removed were performed on log-transformed data, and analyses of jasmonic acid concentration were performed on square

root-transformed data to meet the assumptions of normality and equal variance. *P-value* with an asterisk indicates significant difference ($P \leq 0.05$).

Supplementary Table 2

Individual comparisons for sensitivity experiment

	**Leaf tissue removed (mm^2)**		**JA Concentration ($ng\ g^{-1}$) 1 h damage**		**JA Concentration ($ng\ g^{-1}$) 24 h damage**	
**Treatment Comparison**	**Difference in Means**	***P*-value**	**Difference in Means**	***P*-value**	**Difference in Means**	***P*-value**
Control / 100%	449.7	0.0001*	-150.0	0.001*	-390.4	0.0000001*
Control / 1%	233.5	0.17	-103.9	0.04*	-74.0	0.04*
Control / 10%	384.4	0.002*	-170.6	0.0007*	-142.0	0.0004*
100% / 1%	-216.2	0.04*	46.1	0.49	316.5	0.00003*
100% / 10%	-65.3	0.76	-20.6	0.98	248.4	0.003*
1% / 10%	150.9	0.25	-66.7	0.31	-68.1	0.32

Tukey post hoc multiple comparisons from the feeding assay and jasmonic acid induction experiment with different concentrations of *E,S*-conophthorin (100% dose, 10% dilution, 1% dilution, and solvent control ($n = 8$). The "Difference in Means" values are for non-transformed data. Statistical analyses were performed on square root-transformed data to meet the assumptions of normality and equal variance. *P-value* with an asterisk indicates significant difference ($P \leq 0.05$).
